# Robot‐assisted therapy for upper‐limb rehabilitation in subacute stroke patients: A systematic review and meta‐analysis

**DOI:** 10.1002/brb3.1742

**Published:** 2020-06-26

**Authors:** Wai‐tong Chien, Yuen‐yu Chong, Man‐kei Tse, Cheuk‐woon Chien, Ho‐yu Cheng

**Affiliations:** ^1^ The Nethersole School of Nursing The Chinese University of Hong Kond New Territories Hong Kong; ^2^ University of Bradford Bradford UK

**Keywords:** meta‐analysis, rehabilitation, robot‐assisted therapy, stroke, sub‐acute

## Abstract

**Background:**

Stroke survivors often experience upper‐limb motor deficits and achieve limited motor recovery within six months after the onset of stroke. We aimed to systematically review the effects of robot‐assisted therapy (RT) in comparison to usual care on the functional and health outcomes of subacute stroke survivors.

**Methods:**

Randomized controlled trials (RCTs) published between January 1, 2000 and December 31, 2019 were identified from six electronic databases. Pooled estimates of standardized mean differences for five outcomes, including motor control (primary outcome), functional independence, upper extremity performance, muscle tone, and quality of life were derived by random effects meta‐analyses. Assessments of risk of bias in the included RCTs and the quality of evidence for every individual outcomes were conducted following the guidelines of the Cochrane Collaboration.

**Results:**

Eleven RCTs involving 493 participants were included for review. At post‐treatment, the effects of RT when compared to usual care on motor control, functional independence, upper extremity performance, muscle tone, and quality of life were nonsignificant (all *p*s ranged .16 to .86). The quality of this evidence was generally rated as low‐to‐moderate. Less than three RCTs assessed the treatment effects beyond post‐treatment and the results remained nonsignificant.

**Conclusion:**

Robot‐assisted therapy produced benefits similar, but not significantly superior, to those from usual care for improving functioning and disability in patients diagnosed with stroke within six months. Apart from using head‐to‐head comparison to determine the effects of RT in subacute stroke survivors, future studies may explore the possibility of conducting noninferiority or equivalence trials, given that the less labor‐intensive RT may offer important advantages over currently available standard care, in terms of improved convenience, better adherence, and lower manpower cost.

## Graphical abstract

PRISMA flow chart of study selection. Articles included in the review met all inclusion criteria and with satisfactory methodological validity and clarity (*n* = 11).

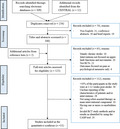

## 1 INTRODUCTION

Stroke is one of the leading causes of death and disability worldwide (Feigin, Lawes, Bennett, & Anderson, 2003). About 17%–40% of stroke survivors experienced upper extremity spasticity, worsening their abilities in performing activities of daily living (ADL) (Hsieh et al., 2017; Pollock et al., 2014). Upper‐limb rehabilitation is crucial during the first six months since the onset of stroke because the motor and ADL recovery of stroke survivors declines afterward (Kwakkel & Kollen, 2013). After the 6‐month poststroke period, 33%– 66% of patients fail to achieve upper‐limb functional recovery (Kwakkel & Kollen, 2013).

Conventional poststroke rehabilitation, including “hands‐on” therapy (manual therapy techniques), constraint‐induced movement therapy, repetitive task training, and mirror therapy (Pollock et al., 2014), usually requires patients to perform partial or full‐assisted movement in arm/hand joints manually under the supervision of therapists. However, the time‐consuming and labor‐intensive nature of conventional therapies has limited its cost‐effectiveness. Robot‐assisted therapy (RT) is a novel approach to poststroke rehabilitation, which utilizes robotic devices to deliver motor or task‐oriented training to patients (Brewer, McDowell, & Worthen‐Chaudhari, 2007). Apart from providing repetitive and high‐intensive training in a cost‐effective fashion (Lo, Stephenson, & Lockwood, 2018), stroke survivors can perform independent training with less supervision from therapists, receive timely feedback on their performance from robotic devices, and achieve better adherence to treatment with an introduction of games or interactive upper‐limb tasks (Hesse, Hess, Werner, Kabbert, & Buschfort, 2014; Kwakkel, Kollen, & Krebs, 2008).

Despite several advantages of RT suggested in literature, there are no conclusive evidence for the beneficial effects of RT over usual care in stroke patients. Two meta‐analyses have indicated that when the dose of RT was matched with that of usual care, no significant between‐group differences were found in motor control and abilities in performing basic ADL (Kwakkel et al., 2008; Norouzi‐Gheidari, Archambault, & Fung, 2012). However, other meta‐analyses have shown that using RT as an adjunct to usual care is more effective than RT alone on improving upper‐limb motor function, in terms of motor control (e.g., Fugl‐Meyer Assessment of the arm), muscle strength/tone and basic ADL (Bertani et al., 2017; Mehrholz, Pohl, Platz, Kugler, & Elsner, 2018; Veerbeek, Langbroek‐Amersfoort, van Wegen, Meskers, & Kwakkel, 2017; Zhang, Li‐Tsang, & Au, 2017). Of note, in the aforementioned reviews (Bertani et al., 2017; Mehrholz et al., 2018; Veerbeek et al., 2017; Zhang et al., 2017), pooling outcomes of RT studies to obtain an overall effect irrespective of different phases of poststroke recovery may have produced overgeneralized conclusions. Throughout the stroke trajectory, patients’ training needs and progress of recovery change over time and may have masked the pooled effect of RT. Though the optimal stroke recovery occurs in the first few months after a stroke, the impacts of RT within six months poststroke remains unclear in literature. In this review, we aimed to examine the research evidence in the past 20 years regarding the effects of RT on outcomes related to body function, activities, and social participation in patients diagnosed with stroke within six months and assess the methodological quality of the included studies.

## 2 METHODS

This review adhered to the Preferred Reporting Items for Systematic Reviews and Meta‐Analyses (PRISMA) guidelines for reporting systematic reviews (Moher, Liberati, Tetzlaff, & Altman, 2009).

### 2.1 Search strategy

We identified studies published between January 1, 2000 and December 31, 2019 in these electronic databases: CINAHL, MEDLINE, PubMed, Embase, SPORT Discus, and Physiotherapy Evidence Database. Additional records were identified from the Cochrane Central Register of Controlled Trials (CENTRAL). Apart from using a Medical Subject Headings term “Stroke Rehabilitation” for searching, we used other keywords related to stroke, RT, and study design according to the PICOS (**P**opulation, **I**ntervention, **C**omparison, **O**utcome, and **S**tudy setting/design) framework (see Appendix S1).

### 2.2 Study selection

Two authors (WTC, HYC) independently reviewed the title and abstract of identified studies, examined the full‐text reports of all potentially relevant studies according to the predefined eligibility criteria. Disagreements of study selection were resolved through discussion with another author (YYC).

We included randomized controlled trials (RCTs) published in English, which had examined the effects of RT. In these RCTs, the participants were at aged 18–65 years (of both gender) in which at least 60% of them had a primary diagnosis of first‐ever stroke with the poststroke period equal or less than six months at the baseline of the study. The included RCTs might adopt RT with different formats (robot‐integrated physiotherapy, home‐based robotic tele‐rehabilitation, robotic training combined with games, and bilateral robotic priming) as the main component of intervention. The RT was reported as a stand‐alone therapy or as an adjunct to conventional therapy used in usual care, while the control conditions could be of any types but not RT, such as conventional therapy and physical therapy. The primary outcome was motor control under the “body function” domain based on the International Classification of Functioning, Disability and Health (ICF) (Sivan, O'Connor, Makower, Levesley, & Bhakta, 2011). The secondary outcomes were functional independence and upper extremity performance from the “activities” domain, muscle tone from the “body function” domain and quality of life (QoL) from the “participation” domain of the ICF. We included studies if they measured at least one outcome under the “body function” or “activities” domain (Sivan et al., 2011).

We excluded studies if: (a) using RT as an adjunct component to other therapy/intervention (except for usual care); (b) the diagnosis of participants were not clearly described, and/or >50% of participants were comorbid with other injuries, surgical interventions, and/or serious upper‐limb impairments, thereby being undesirable to perform upper‐limb training.

### 2.3 Data extraction

Two authors (MKT, CWC) independently extracted the following information of each included study: study design, characteristics of the participants, treatment conditions in both arms, outcome measures/instruments, main findings, attrition rates, and safety and cost of RT, using a self‐developed data extraction form. Disagreements of data extraction were resolved through discussion with another author (YYC).

### 2.4 Risk of bias assessments of included studies

Two authors (WTC, MKT) independently assessed the overall risk of bias of the included studies by using the Revised Cochrane risk of bias tool (RoB2) (Sterne et al., 2019). The RoB2 covers five domains of bias, including bias arising from randomization; bias due to deviations from intended interventions; bias due to missing outcome data; bias in measurement of outcome; and bias in selection of the reported result. An overall risk of bias judgement was rated for each included study, ranking from low, some concerns to high risk of bias. The authors resolved the disagreements of assessments with another author (YYC) by discussion.

### 2.5 Data analysis

For each included study reported with continuous data, we calculated the between‐group effect sizes (ESs) by comparing the means between groups at postintervention (<3, 3–7 and >7 months postintervention) using the following formula (Cohen, 1988): *d* = *M*_1_–*M*_2_/*SD*_pooled_ (*M*_1_ and *M*_2_ refer to the means of both groups). The pooled standard deviation (*SD*_pooled_) was calculated by using the following formula (Cohen, 1988): *SD*_pooled_ = √ (*SD*_1_^2^ + *SD*_2_^2^)/2. Next, for the outcome being assessed by more than two RCTs, we calculated the pooled effects using SMDs with 95% confidence intervals (CIs) through random effects models accounting for the variations in the use of instruments. When standard errors (*SE*s), but not *SD*s, were available, we computed the missing SDs by using the following formula: *SD* = *SE*√*n* (Higgins & Green, 2011). To avoid unit of analysis error in three‐arm RCTs, the continuous outcomes of two intervention groups were combined into a single intervention group and compared this with the results of the control group. The magnitude of SMDs can be interpreted as small (0.2), medium (0.5), or large (0.8) (Cohen, 1988). In addition, we assessed the heterogeneity between RCTs by using the *I* ^2^ statistic, an *I* ^2^ larger than 50% with *p* < .05 indicates a large and significant heterogeneity across studies. We used the RevMan 5.3 software to conduct the aforementioned analyses. If the studies reported with skewed data as medians and interquartile ranges, or if there is a lack of RCTs (≤2 per outcome), we excluded them from meta‐analyses.

### 2.6 Quality of evidence

We assessed the quality of evidence as high, moderate, low, or very low based on the Grading of Recommendations Assessment, Development and Evaluation (GRADE) guidelines (GRADE Working Group, 2013). A summary of findings (SoF) table was generated by the GRADEpro GDT (GRADEpro Guideline Development Tool, 2015) to present the magnitude of effect of RT in comparison with usual care for primary and secondary outcomes. In the SoF, we followed the recommendations from the Cochrane Collaborations and presented the magnitude of effects for motor control and functional independence by re‐expressing the SMDs generated from the pooled data into the mean differences (MDs) (Higgins & Green, 2011).

### 2.7 Ethical statement

This article does not contain any studies with human participants or animals performed by any of the authors; thus, ethical approval is not required.

## 3 RESULTS

**FIGURE 1:**
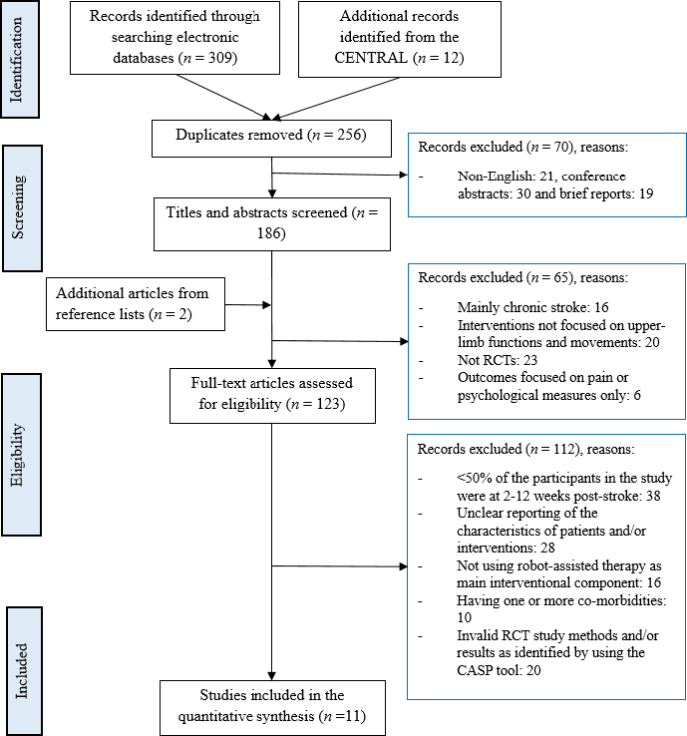
PRISMA flow chart of study selection

Our search yielded a total of 321 records. After removing duplicates, non‐English and brief reports, 186 abstracts were screened. Two additional articles were identified from the reference lists. After removing noneligible studies and studies reporting invalid methods and/or results in accordance to the Critical Appraisal Skills Programme criteria (Critical Appraisal Skills Programme, 2018), eleven RCTs were included in this review (Barker, Hayward, Carson, Lloyd, & Brauer, 2017; Daunoraviciene, Adomaviciene, Grigonyte, Griskevicius, & Juocevicius, 2018; Dehem et al., 2019; Hesse et al., 2014; Masiero, Armani, Ferlini, Rosati, & Rossi, 2014; Orihuela‐Espina et al., 2016; Sale et al., 2014; Stinear, Petoe, Anwar, Barber, & Byblow, 2014; Villafane et al., 2018; Volpe et al., 2000; Wolf et al., 2015) (see Figure 1).

### 3.1 Included studies

Table 1 presents the characteristics of these studies.

**TABLE 1:** Characteristics of the included studies

Study (Country)	Design and assessment time points	Participants	Characteristics of the participants	Intervention	Instruments	Key finding(s)	Attrition rate	
Barker 2017 (Australia)	3‐arm RCT in repeated measures design, at baseline, post‐treatment, 26 and 52 weeks post‐treatment	Consecutively admitted patients at one acute stroke unit (*n* = 50) were randomized into 3 groups using random permuted blocks	Mean age = 53.6 ± 15 Male = 68% Stroke intervals = 5.9 ± 3 weeks	Treatment groups SMART Arm^b^ training with stimulation for elbow, forearm, wrist, and hand plus conventional therapy (*n* = 17) SMART Arm^b^ training without stimulation plus conventional therapy (*n* = 16) Both groups received 1‐hr session on weekdays for 4 weeks Content: perform reaching task in a straight‐line path. Control group ( *n* = 17) 1‐hr conventional therapy, including physiotherapy, occupational therapy, and therapy assistant time, and involved a mix of one‐on‐one and group therapy sessions on weekdays for 4 weeks	MAS –item 6^a^ MRC MAS RAI MAS –item 6,7,8 SIS MAL kinematics and kinetics of reaching.	The RT group achieved better upper arm function when compared to the control group at post‐training (OR = 1.47, 95% CI = 1.23–1.71) and at 26 weeks (OR = 1.31, 95% CI = 1.05–1.57), respectively. There was no significant between‐group difference in arm function (*p* = .37). All groups showed significant improvements in arm function and quality of life (all *p*s < 0.001) over time. No significant improvements in muscle tone among all groups at any assessment time points.	Post‐treatment = 6%; 26 weeks post‐treatment = 22%; 52 weeks post‐treatment = 30%	
Daunoraviciene 2018 (Lithuania)	2‐arm RCT in pre‐post design	Subacute stroke patients (*n* = 34) were randomly assigned into 2 groups (Sampling strategies not reported)	Mean age = 65.7 ± 4.48 Male = 65% Stroke intervals = 9.1 ± 5 weeks	Treatment group ( *n* = 17) RT with Armeo Spring^c^ for elbow, forearm, wrist, and hand for 1‐hr session on weekdays for 2 weeks Content: perform a sequence of motor tasks in 5–7 exercise cycles Control group ( *n* = 17) Occupational therapy sessions, including exercising, physical activities, active table games etc.	FIM‐self‐care^a^ FMA HAM‐D HAM‐A ACE‐R MAS Active ROM	The RT group showed significantly better functional independence when compared with the UT group at postintervention (*p* < .03). No significant between‐group difference in motor control at postintervention (*p* = .287)	No attrition was found at postintervention	
Dehem 2019 (Belgium)	2‐arm RCT in repeated measures design at baseline, postintervention, and 6‐month poststroke	Subacute stroke patients (*n* = 45) with < 1 month poststroke from inpatient rehabilitation centers were randomized into two groups using computer‐generated sequence	Mean age = 67.9 ± 15.4 Male % = 46.7% Stroke intervals = 27.8 ± 5.5 days	Treatment group ( *n* = 23) Four 45‐min RT sessions for wrist and hand with REAplan robot^d^ (25%) and twelve UT sessions (75%) per week for 9 weeks Content: exercises with game involving moving the paretic hand along a reference trajectory while passing through checkpoints Control group ( *n* = 22) Sixteen 45‐min UT session per week for 9 weeks Content: motor rehabilitation that matched with patients’ personal needs	FMA‐UE^a^ BBT WMFT ABILHANDACTIVLIM SIS	RT showed significantly greater improvement in gross manual dexterity (*p* = .02), upper‐limb ability (*p* =.02) and patient social participation (*p* = .01) compared with control group at six months poststroke Both group show similar improvement in abilities to perform manual activities and activities of daily living	Postintervention = 28.9%, six months poststroke = 37.8%	
Hesse 2014 (Germany)	2‐arm RCT in repeated measures design at baseline, post‐treatment and three‐month post‐treatment	Subacute patients from two inpatient stroke rehabilitation units (*n* = 50) were assigned into 2 groups by web‐based randomization tool	Mean age = 70.6 ± 16.1 Male = 56% Stroke intervals = 4.5 ± 2 weeks	Treatment group ( *n* = 25) 30‐min of RT in arm studio plus 30‐min individual arm therapy on weekdays for 4 weeks Content: 1) repetitive practice of finger, wrist, forearm, and shoulder movement; 2) task‐oriented motor relearning program and impairment‐oriented arm ability training Control group ( *n* = 25) 1‐hr individual arm therapy on weekdays for 4 weeks, consisting of the task‐oriented motor relearning program and the impairment‐oriented arm ability training (repetitions of movements and shaping)	FMA^a^ ARAT BBT MRC MAS BI	No significant between‐group differences were found in motor control, upper extremity performance, muscle tone, and functional independence at all measurement points. Both groups showed significant improvements in motor control and upper extremity performance at post‐training and at three‐month follow‐up (all *p*s <0.001)	Post‐treatment = 2%, 3 months post‐treatment = 8%	
Masiero 2014 (Italy)	2‐arm RCT in repeated measures design at baseline, post‐treatment, 3 months and 7 months post‐treatment	Subacute subjects from Stroke Unit (*n* = 34) were randomly allocated to 2 groups by a computer program	Mean age = 66.3 ± 8.55 Male = 66.7% Stroke intervals = 1.3 ± 0.4 week	Treatment group ( *n* = 16) ~75‐min of standard therapy plus ~ 45‐min of RT for elbow, foreman, wrist, and hand on weekday for 5 weeks Content: 1) NeReBot training^e^ with exercises (flexion and extension, adduction and abduction, pronation and supination, circumduction); 2) Conventional functional rehabilitation including proprioceptive exercises, functional reduction, gait training, occupational therapy, passive, and active assisted mobilization of the hand, wrist, and upper paretic arm Control group ( *n* = 18) 2‐hr of daily rehabilitation treatment for 5 weeks including proprioceptive exercises, functional re‐education, gait training, occupational therapy, and passive and active assisted mobilization of the hand and wrist.	FMA^a^ FIM‐motor^a^ FAT^a^ MRC MAS BBT tolerability and acceptability of treatment	No significant between‐group differences were found in motor function, muscle tone, and functional independence at all assessment time‐points.		At post‐treatment = 11.8%; 7 months post‐treatment = 17.6%
Orihulela Espina 2016 (Mexico)	2‐arm RCT in pre‐post design	Subacute stroke patients from Neurologic Rehabilitation Unit (*n* = 17) were randomly assigned into 2 groups by block randomization	Mean age = 55.6 ± 20.3 Male = 64.7% Stroke intervals = 1 week to 4 months	Treatment group ( *n* = 9) 1‐hr of RT for wrist and hand on weekdays for around 8 weeks Content: 1) passive activities; 2) partial assistance/ resistance activities; 3) active movement Control group ( *n* = 8) 1‐hr of classical occupational therapy on weekdays for around 8 weeks Content: massage and conventional occupational exercises, including passive movements, strengthening exercises and active grasps movement and personalized activities for fine pinching control	FMA‐hand^a^ MI^a^	RT showed significantly greater improvement in hand motor function compared with UT at post‐treatment (*p* < .01). Both groups showed significant improvement in motor control over time (Nonparametric Cliff's delta‐within effect sizes: dwOT‐FMA = 0.5, dwRT‐FMA = 1)		No attrition was found at postintervention
Sale 2014 (Italy)	2‐arm RCT in repeated measures design at baseline, after 15^th^ and 30^th^ treatment sessions	Subacute stroke patients from the rehabilitation center (*n* = 53) were randomly allocated into 2 groups by dedicated software	Mean age = 67.7 ± 14.2 Male = 58.5% Stroke intervals = 4.3 ± 1week	Treatment group ( *n* = 26) 45‐min of RT with MIT‐MANUS^h^ for shoulder and elbow plus 3‐hr physiotherapy on weekdays for 6 weeks Content: 1) dexterity and gait training, 2) goal‐directed, planar reaching tasks, including both unassisted and assisted repetitions Control group ( *n* = 27) 45‐min of conventional therapy plus 3‐hr of physiotherapy on weekdays for 6 weeks Content: 1) dexterity and gait training; 2) assisted stretching, shoulder and arm exercises and functional reaching tasks exercising, physical activities, and active table games	FMA^a^ MAS‐S^a^ MAS‐E^a^ pROM MI	Both groups showed significant improvements in motor control after 15^th^ and 30^th^ session, with significant greater improvement found in the RT group after the first 15^th^ sessions (*p* <.0001). Significant improvement in muscle tone for shoulder and elbow (*p* <.05) was only found in the RT group.		No attrition was found after 30^th^ treatment sessions
Stinear 2014 (New Zealand)	2‐arm RCT in repeated measures design at baseline, 6, 12 and 26 weeks post‐treatment	Consecutive subacute stroke patients from a stroke unit (*n* = 57) were randomized by customized software (www.rando.la)	Mean age = 68 ± 25 Male = 45.6% Stroke intervals < 26 days	Treatment group ( *n* = 29) 15‐min Bilateral priming for wrists and hands plus 30‐min physiotherapy and occupational therapy on weekdays for 4 weeks Control group ( *n* = 28) 15‐min Intermittent cutaneous electric stimulation plus 30‐min physiotherapy and occupational therapy on weekdays for 4 weeks	ARAT^a^ SIS MRS	At 12^th^ weeks, greater proportion of the participants in the treatment group achieved their recovery plateaus in upper extremity performance than the control group (χ^2^ = 4.25; *p* = .039). At 26^th^ weeks, no between‐group difference was found in quality of life^5^		At post‐treatment = 7.0% At 12 weeks post‐treatment = 10.5% At 26 weeks post‐treatment = 15.8%
Villafane 2018 (Italy)	2‐arm RCT in pre‐post design	Acute stroke patients (*n* = 32) with hand paralysis from rehabilitation hospitals were randomized into two groups using simple randomization	Mean age = 68.9 ± 11.6 Male% = 65.6% Stroke intervals: < 3 moths	Treatment group ( *n* = 16) 1hr physical and occupational therapy on weekdays + 30‐min RT on 3 days per week for three weeks Content: passive mobilization of hand through robotic device Gloreha^f^ Control group ( *n* = 16) 1hr physical and occupation therapy + 30 min standard rehabilitation on 3 days per week for three weeks Content: assisted stretching, shoulder, and arm exercises and functional reaching tasks	NIHSS^a^ MAS BI MI QuickDASH VAS	RT showed greater reduction in pain compared with UT at postintervention (Cohen's d = 1.73) Except MAS, NIHSS, BI, MI, and QuickDASH showed improvement in both group at post‐treatment ( *p* <.001)		No attrition was found at post‐treatment
Volpe 2000 (USA)	2‐arm RCT in pre‐post design	Subacute patients from inpatient rehabilitation stroke unit (*n* = 56) was randomly assigned into 2 groups (Sampling strategies not reported)	Mean age = 64.3 ± 3.20 Male = 53.4% Stroke intervals = 2.1 ± 0.2 weeks Baseline FMA‐ shoulder and elbow *M* = 5.54 *SD* = 2.01	Treatment group ( *n* = 30) Standard physical and occupational poststroke therapy plus 1‐hr RT per day with MIT‐MANUS^h^ on weekdays for 5 weeks Content: >1,500 repetitions of goal‐directed shoulder, elbow, wrist, and hand movement to a target Control group ( *n* = 26) Standard physical and occupational poststroke therapy plus 1‐hr per week of exposure to the robot without training	FMA‐SEC^a^ FMA‐WH^a^ MS‐SE MS‐WH MP FIM‐Motor FIM‐Cognition	The RT group showed significantly better functional independence compared to the UT group at post‐treatment (*p* < .01). No significant between‐group difference was found in motor control.		No attrition was found at post‐treatment
Wolf 2015 (USA)	2‐arm RCT in pre‐post design	Subacute stroke patients (*n* = 99) were randomly assigned into 2 groups using a stratified, computer‐driven randomization procedure	Mean age = 57.0 ± 13.4 Male = 56.6% Stroke intervals = 17.1 ± 7 weeks	Treatment group ( *n* = 51) 3‐hr session including RT with the Hand Mentor Pro (HMP)^g^ and home exercise program on weekdays for 8–12 weeks Content: 1) Wrist and fingers exercises; 2) functional activity Control group ( *n* = 48) 3‐hr of home exercise program on weekdays for 8–12 weeks Content: 1) Traditional impairment‐based activities, for example, weight‐bearing activates, active assisted exercises, shoulder exercises etc.; 2) functional activities	ARAT^a^ WFMT FMA	No significant between‐group difference was found in motor control and upper extremity performance. Both groups showed improvement in motor control and upper extremity performance over time (all *p*s < 0.001)		7.1%

Abbreviations: ACE‐R, Addenbrooke Cognitive Examination‐Revised; Active ROM, Active Range of Motion; ARAT, Action Research Arm Test; BBT, Box and Block Test; BI, Barthel Index; FAT, Frenchay Arm Test; FIM, Functional Independence Measurement; FMA, Fugl‐Meyer Assessment; HAM‐A, Hamilton Rating Scale for Anxiety; HAM‐D, Hamilton Rating Scale for Depression; MAL, Motor Activity Log‐28; MAS, Modified Ashworth Scale; MAS, Motor Assessment Scale; MI, Motricity Index; MP, Motor Power Scale; MRC, Medical Research Council, MRS, Modified Rankin Scale; MS, Motor Status Score; NIHSS, the National Institutes of Health Stroke Scale; pROM, passive Range of motion; QuickDASH, short version of the Disabilities of the Arm, Shoulder and Hand; RAI, Ritchie Articular Index; RCT, randomized controlled trial; RT, Robot‐assisted therapy; SIS, Stroke Impact Scale; VAS, Visual Analog Scale; WFMT, Wolf Motor Function Test.

^a^ Primary outcome(s) of the included study.

^b^ SMART ArmTM http://smartarm.com.au/development/.

^c^ Armeo Spring: https://www.hocoma.com/solutions/armeo‐spring/.

^d^ REAplan robot https://www.axinesis.com/en/.

^e^ Masiero, S., Celia, A., Armani, M., & Rosati, G. (2006). A novel robot device in rehabilitation of post‐stroke hemiplegic upper limbs. Aging clinical and experimental research, 18(6), 531–535.

^f^ Gloreha https://www.gloreha.com.

^g^ The Hand Mentor Pro (HMP) https://motusnova.com/products/hand‐mentor‐pr.

^h^ MIT‐MANUS/ InMotion2, (Interactive Motion Technologies, Inc., Watertown., MA, USA).

#### 3.1.1 Design and participants

Of the 11 included RCTs (493 participants), six included follow‐up assessment(s) (Barker et al., 2017; Dehem et al., 2019; Hesse et al., 2014; Masiero et al., 2014; Sale et al., 2014; Stinear et al., 2014), and five reported the assessments at post‐treatment only (Daunoraviciene et al., 2018; Orihuela‐Espina et al., 2016; Villafane et al., 2018; Volpe et al., 2000; Wolf et al., 2015). One study assessed the outcomes after the 15th and 30th sessions (Sale et al., 2014), the remaining studies measured the outcomes starting from immediately to 52 weeks postintervention (*M* = 26.0, *SD* = 14.6). The stroke survivors (age: *M* range = 54–71, *SD* range = 3.2–25.0, male: 46%–68%) were recruited from stroke units or inpatient rehabilitation centers mainly in Western countries. The mean duration from the stroke onset to study entry was 6 weeks (*SD* = 4.7, range = 9 days–4 months).

#### 3.1.2 Robot‐assisted therapy

Seven studies integrated RT with usual care and the time spent for RT in proportion to the whole treatment varied from 25%–50% (Barker et al., 2017; Dehem et al., 2019; Hesse et al., 2014; Masiero et al., 2014; Stinear et al., 2014; Villafane et al., 2018; Wolf et al., 2015). The remaining four studies provided RT as a stand‐alone treatment (Daunoraviciene et al., 2018; Orihuela‐Espina et al., 2016; Sale et al., 2014; Volpe et al., 2000). The number of sessions for RT ranged from 9–40 (*M* = 25.0, *SD* = 10.2), with each session lasted for 30–120 min (*M* = 75.0, *SD* = 35.2). Participants received RT for five days per week for 2–12 weeks (*M* = 5.6, *SD* = 2.8). Eight studies were dose‐matched RCTs in which the participants in the assigned groups received identical duration of treatment (Barker et al., 2017; Hesse et al., 2014; Masiero et al., 2014; Orihuela‐Espina et al., 2016; Sale et al., 2014; Stinear et al., 2014; Villafane et al., 2018; Wolf et al., 2015). Two used MIT‐MANUS (Sale et al., 2014; Volpe et al., 2000). All studies offered RT through a single robot device, except Hesse et al. (Hesse et al., 2014) in which the participants received training of finger, wrist, forearm, and shoulder by six different robotic devices. Of the included studies, RT targeted different areas of the upper limb, including wrist and hand only (Dehem et al., 2019; Orihuela‐Espina et al., 2016; Stinear et al., 2014; Villafane et al., 2018; Wolf et al., 2015), the whole upper limb (elbow, foreman, wrist, and hand) (Barker et al., 2017; Daunoraviciene et al., 2018; Masiero et al., 2014), shoulder, and elbow only (Sale et al., 2014), the whole upper limb plus shoulder (Hesse et al., 2014; Volpe et al., 2000). The mean attrition rate was 10.5% (range = 0%–28.9%). All studies reported with no serious adverse events, but one study reported that nine patients experienced discomfort and two patients had blisters in the fingertips after RT (Hesse et al., 2014). The costs per patient were 4.15 € for RT and 10.00 € for conventional therapy, respectively (Hesse et al., 2014).

#### 3.1.3 Control conditions

All included studies adopted usual care as control condition, including physiotherapy (Barker et al., 2017; Sale et al., 2014; Wolf et al., 2015), occupational therapy (Daunoraviciene et al., 2018; Orihuela‐Espina et al., 2016; Volpe et al., 2000), task‐orientated/impairment‐oriented arm training program (Hesse et al., 2014; Villafane et al., 2018), daily rehabilitation treatment (Dehem et al., 2019; Masiero et al., 2014), intermittent cutaneous electric stimulation (Stinear et al., 2014), and home exercise program (Wolf et al., 2015), respectively.

#### 3.1.4 Outcome measures

Motor control was measured by the Fugl‐Meyer Assessment (FMA) (Daunoraviciene et al., 2018; Sale et al., 2014; Wolf et al., 2015) and its related scales, including the FMA‐motor (Hesse et al., 2014), the FMA for shoulder/elbow and coordination (FMA‐SEC) (Volpe et al., 2000) and the FMA for wrist and hand function (FMA‐WH) (Orihuela‐Espina et al., 2016). Functional independence was measured by the Functional Independence Measures (FIM)‐self‐care domain (Daunoraviciene et al., 2018), the FIM‐motor (Volpe et al., 2000), the FIM‐cognition (Volpe et al., 2000), the Barthel Index (BI) (Hesse et al., 2014) and the Activlim questionnaire (Dehem et al., 2019). Of the included RCTs, three applied the Action Research Arm Test (ARAT) (Hesse et al., 2014; Stinear et al., 2014; Wolf et al., 2015), two used the Wolf Motor Function Test (WMFT) (Wolf et al., 2015) and one used QuickDASH (Villafane et al., 2018) to assess upper extremity performance; six used the Modified Ashworth Scale (MAS) to assess muscle tone (Barker et al., 2017; Daunoraviciene et al., 2018; Hesse et al., 2014; Masiero et al., 2014; Sale et al., 2014; Villafane et al., 2018); and three used the Stroke Impact Scale (SIS) to measure quality of life (Barker et al., 2017; Dehem et al., 2019; Stinear et al., 2014).

### 3.2 Risk of bias

**FIGURE 2:**
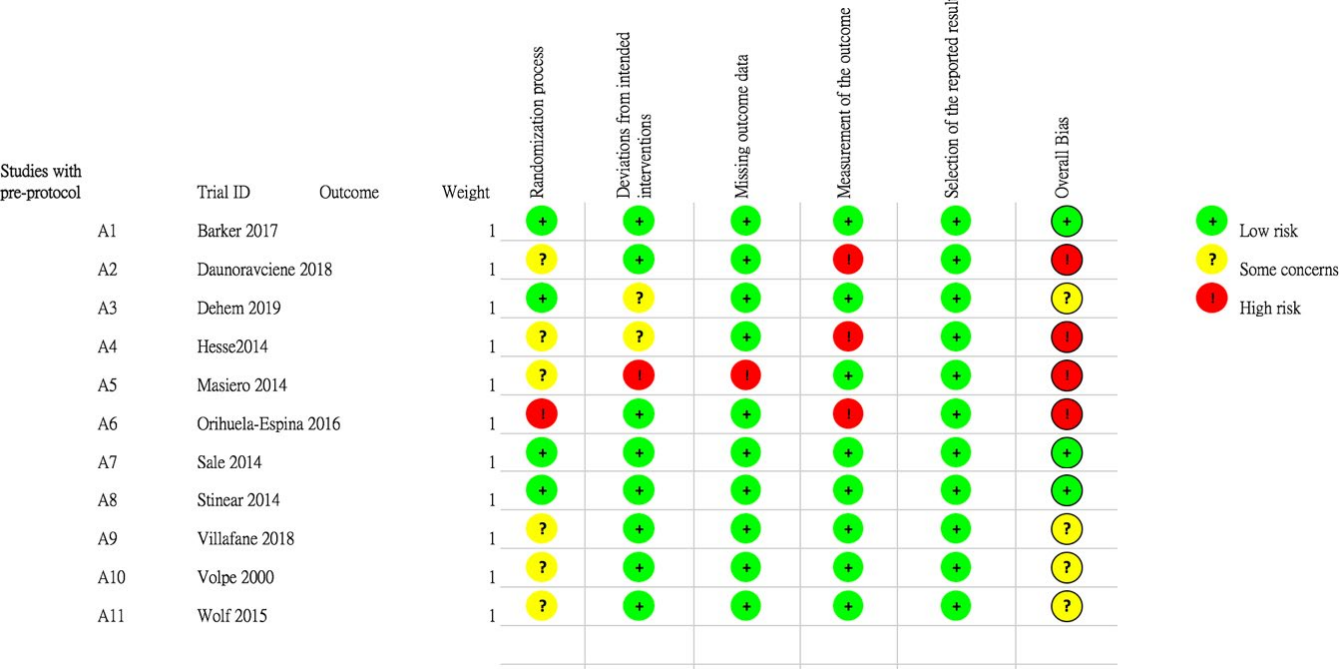
Summary of risk of bias of each included study

**FIGURE 3:**
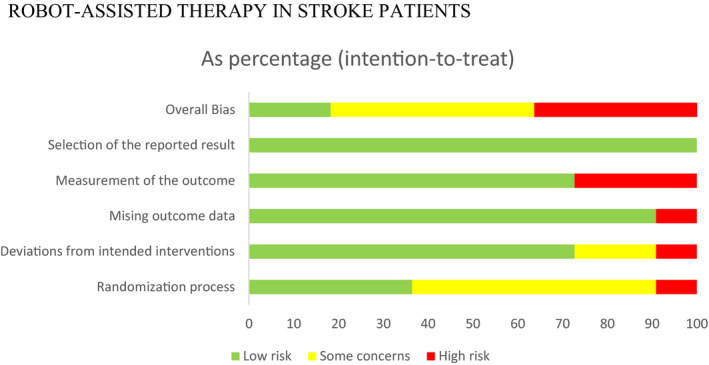
Summary of risk of bias across all included studies

Four included RCTs were considered as “high risk” of overall bias (Daunoraviciene et al., 2018; Hesse et al., 2014; Masiero et al., 2014; Orihuela‐Espina et al., 2016), four were rated as “some concerns” (Volpe et al., 2000; Wolf et al., 2015), and three were judged as “low risk” (Barker et al., 2017; Sale et al., 2014; Stinear et al., 2014) (see Figures 2 and 3). Specifically, six RCTs described random sequence generation with insufficient information on how the allocation was concealed (Daunoraviciene et al., 2018; Hesse et al., 2014; Masiero et al., 2014; Volpe et al., 2000; Wolf et al., 2015); and one study did not implement allocation concealment and was judged as at high risk of bias (Orihuela‐Espina et al., 2016). Bias due to deviations from the assigned interventions and missing outcome data were relatively low across studies, given that the attrition mainly occurred in the conventional therapy or usual care (e.g., hospital discharge). Attrition rates at post‐treatment (0%–28.9%) and follow‐up (0%–18%) were low across studies.

### 3.3 Effects of robotic‐assisted therapy

The effect sizes of RT on the primary and secondary outcomes of each included RCT were tabulated in Appendix S2.

#### 3.3.1 Motor control

**FIGURE 4:**
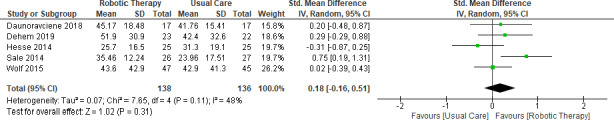
Forest plot: Comparison of the effect of robotic‐assisted therapy and usual care on motor control at post‐treatment

At post‐treatment, the overall effect of RT for improving motor control was insignificant when compared to usual care (SMD = 0.18, 95%CI −0.16, 0.51*, p* = .31, 5 RCTs, 274 participants) (see Figure 4). As shown in the SoF, the quality of evidence using the GRADE approach was downgraded from high to low (2 points) because two of the pooled studies were considered at high risk of bias (Daunoraviciene et al., 2018; Hesse et al., 2014), and the pooled effect was based on wide 95% confidence interval (see Appendix S3) (Daunoraviciene et al., 2018; Dehem et al., 2019; Hesse et al., 2014; Sale et al., 2014; Wolf et al., 2015). Hesse et al. (2014) assessed the treatment effect at 3‐month post‐intervention, but no significant effect was found.

#### 3.3.2 Functional independence

**FIGURE 5:**
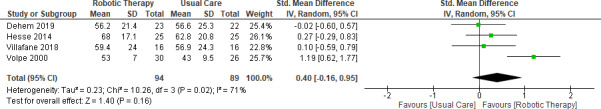
Forest plot: Comparison of the effect of robotic‐assisted therapy and usual care on functional independence at post‐treatment

At post‐treatment, the overall effect of RT for improving functional independence was not significant when compared to usual care (SMD = 0.40, 95%CI −0.16, 0.95, *p* = .16, 4 RCTs, 183 participants) (see Figure 5) (Dehem et al., 2019; Hesse et al., 2014; Villafane et al., 2018; Volpe et al., 2000). In the SoF, we downgraded the quality of this evidence using the GRADE approach from high to low (2 points) because one of the pooled studies (Hesse et al., 2014) was at high risk of bias and the sample size per arm in each included study was small (16–30 per arm) (Dehem et al., 2019; Hesse et al., 2014; Villafane et al., 2018; Volpe et al., 2000) (see Appendix S3). Dehem et al. (2019) measured the treatment effect at 6‐month poststroke, but no significant effect was found.

#### 3.3.3 Upper extremity performance

**FIGURE 6:**
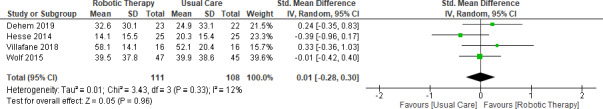
Forest plot: Comparison of the effect of robotic‐assisted therapy and usual care on upper extremity performance at post‐treatment

At post‐treatment, the overall effect of RT for improving upper extremity performance was nonsignificant, when compared to usual care (SMD = 0.01, 95%CI −0.28, 0.3, *p* = .96, 4 RCTs, 219 participants) (see Figure 6) (Dehem et al., 2019; Hesse et al., 2014; Villafane et al., 2018; Volpe et al., 2000). We downgraded the quality of this evidence from high to moderate (1 point) because one of the pooled studies (Hesse et al., 2014) was at high risk of bias (see Appendix S3). Two studies measured the treatment effect at 3‐month (Hesse et al., 2014) and 6‐month (Dehem et al., 2019) poststroke, respectively, but no significant effects were found.

#### 3.3.4 Muscle tone

**FIGURE 7:**
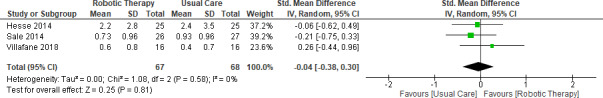
Forest plot: Comparison of the effect of robotic‐assisted therapy and usual care on muscle tone at post‐treatment

The overall effect of RT for improving muscle tone was nonsignificant, when compared to usual care at post‐treatment (SMD = −0.04, 95%CI −0.38, 0.30, 3 RCTs, *p* = .81, 135 participants) (see Figure 7) (Hesse et al., 2014; Sale et al., 2014; Villafane et al., 2018). The quality of this evidence was downgraded from high to low (2 points) because one of the pooled studies (Hesse et al., 2014) was at high risk of bias and the sample size per arm in each included study was small (16–26 per arm; see Appendix S3) (Hesse et al., 2014; Sale et al., 2014; Villafane et al., 2018). Hesse et al. (2014) measured the treatment effect at 6‐month poststroke, but no significant effect was shown.

#### 3.3.5 Quality of life

**FIGURE 8:**
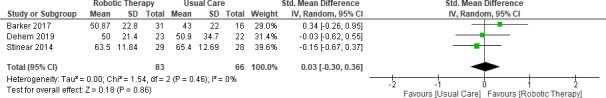
Forest plot: Comparison of the effect of robotic‐assisted therapy and usual care on quality of life at post‐treatment

The overall effect of RT for improving quality of life was not significant, when compared to usual care at post‐treatment (SMD = 0.03, 95%CI −0.30, 0.36, *p* = .86, 3 RCTs, 149 participants) (see Figure 8) (Barker et al., 2017; Dehem et al., 2019; Stinear et al., 2014). We downgraded the quality of this evidence from high to moderate (1 point) because of the small sample size per arm in each included RCT (16–31 per arm, see Appendix S3) (Barker et al., 2017; Dehem et al., 2019; Stinear et al., 2014). Two studies (Barker et al., 2017; Dehem et al., 2019) assessed the treatment effect at 52‐week and 6‐month poststroke, respectively, but no significant effects were found.

## 4 DISCUSSION

We systematically reviewed a total of 11 RCTs to examine whether the effects of RT outweighed usual care for improving motor control, functional independence, upper extremity performance, muscle tone, and quality of life in patients experienced in early stage of poststroke rehabilitation. Nonsignificant effects were found in all our selected outcomes at post‐treatment up to 12‐month post‐treatment and the evidence was generally rated as low‐to‐moderate quality.

The aforementioned findings were in line with a previous systematic review published in 2012, suggesting the effects of RT was not different from that of dose‐matched conventional therapy or usual care regardless the poststroke rehabilitation phase (i.e. acute, subacute, or chronic) (Norouzi‐Gheidari et al., 2012). In the present review, all participants of the included RCTs were enrolled within six months after their first episode of stroke. For those who were allocated to the control conditions, the standard care that they received from physiotherapists and/or occupational therapists were often well‐evidenced and recommended as clinical treatments (Barker et al., 2017; Hesse et al., 2014; Masiero et al., 2014; Orihuela‐Espina et al., 2016; Sale et al., 2014; Stinear et al., 2014; Villafane et al., 2018; Wolf et al., 2015). Hence, when the dose of RT was matched with conventional therapy or usual care or even acted as an adjunct therapy to usual care, the additional benefit of RT for producing high‐intensity movement no longer existed. In other words, the gains in motor and functional outcomes in stroke patients at post‐treatment appeared to be attributed to highly intensive and repetitive movements, regardless of whether it was delivered by therapists or robotic devices. We also found that adverse events were uncommon and the mean attrition rate at postintervention was low (approximately 10%), indicating that RT is generally safe and acceptable to most participants at the subacute phase of stroke. As only one study assessed the cost of RT (Hesse et al., 2014), the cost‐effectiveness of RT remains uncertain.

### 4.1 Study limitations and implications

The small number of included RCTs per outcome and large clinical heterogeneity across trials confined us from meta‐analyzing the effects of RT on our selected outcomes at post‐treatment and follow ups. In addition, most of the included studies were at uncertain or high risk of bias due to insufficient information on allocation concealment and the lack of blinding of outcome assessors. We only included RCTs that were written in English, which may have inadvertently omitted other relevant studies that were published in other languages. Our findings might have limited generalizability to the Asian populations as the reviewed RCTs were conducted in Western countries.

Previous review has indicated the lack of evidence supporting the effects of RT for people affected by stroke within the first three months (Veerbeek et al., 2017). Our findings address this knowledge gap, given that all the participants of the included RCTs were enrolled within six months after their first episode of stroke. Nevertheless, there is still a need for well‐designed, adequately powered RCTs to evaluate the benefits and harms of RT in subacute stroke survivors. Apart from providing more standardized and detailed information regarding the components of RT, trial authors shall specify whether they are adopting an intensity‐matched and/or duration‐matched design for a head‐to‐head comparison with the control arm. In addition, further research may examine the effect of RT tailoring to patients undergoing different phases of stroke recovery/rehabilitation, and standardize the use of parameters for a better comparability between outcomes across studies. For a more comprehensive evaluation of RT, future studies could include more patient‐reported and practical (e.g., safety, adherence, and cost) outcomes (Reeves et al., 2018), explore participants’ experiences in receiving RT and investigate the long‐term effects of RT. Aside from superiority trials to determine whether RT demonstrates better therapeutic effects when compared with usual care, future studies may explore the possibility of conducting noninferiority or equivalence trials, given that the less labor‐intensive RT may offer important advantages over currently available standard care, in terms of improved convenience, better adherence, and lower manpower cost.

## 5 CONCLUSIONS

Robot‐assisted therapy produced benefits similar, but not significantly superior to those from usual care, for improving motor control, functional independence, upper extremity performance, muscle tone, and quality of life in individuals who were diagnosed with stroke within the first six months poststroke at post‐treatment. The therapeutic effects of RT beyond post‐treatment remain insignificant. The aforementioned evidence, based on small number of trials (<5 RCTs) with low methodological quality, should be interpreted with caution.

## CONFLICT OF INTEREST

The authors declare that there are no conflicts of interest.

## AUTHOR CONTRIBUTIONS

Wai‐tong Chien designed this review. Wai‐tong Chien, Yuen‐yu Chong, and Ho‐yu Cheng searched literature. Wai‐tong Chien, Yuen‐yu Chong, Man‐kei Tse, and Cheuk‐woon Chien analyzed the data. Wai‐tong Chien, Yuen‐yu Chong, Man‐kei Tse, and Ho‐yu Cheng wrote the manuscript. All authors approved the final manuscript as submitted.

### Peer Review

The peer review history for this article is available at https://publons.com/publon/10.1002/brb3.1742.

## Supporting information

Supplementary Material

## Data Availability

The data that support the findings of this study are available from the corresponding author upon reasonable request.
