# Supplementary material for: Robot‐assisted therapy for upper‐limb rehabilitation in subacute stroke patients: A systematic review and meta‐analysis
Source: Brain Behav. 2020 Jun 26;10(8):e01742. doi: 10.1002/brb3.1742 (PMC7428503; doi:10.1002/brb3.1742)
Supplement: Supplementary file 1 — Supplementary Material [file BRB3-10-e01742-s001.docx]

**Appendix S1.** Search terms and keywords

| Search free text terms / Keywords |
| --- |
| #1 **MeSH:** Stroke rehabilitation |
| **P*opulation*** |
| *#2* stroke OR subacute stroke OR sub-acute stroke OR CVA OR cerebrovascular accident OR cerebral stroke |
| *#3* upper limb OR arm OR upper extremity OR hand |
| #4 paresis OR paretic OR hemiplegia OR paralysis OR weakness |
| *#5 #2 AND #3 AND #4* |
| **I*ntervention*** |
| *#6* robot* OR robotics OR electro-mechanic OR bioelectronics OR bionic OR Exoskeleton device |
| *#7* rehabilitation OR training OR therapy OR program OR device |
| *#8 #6 AND #7* |
| **C*omparison*** |
| #9 usual care OR treatment-as-usual OR rehabilitation OR upper-limb train* OR standard care |
| **O*utcomes*** |
| #10 function OR impairment OR disability OR movement OR strength OR activity OR ADL |
| **S*tudy design /* S*etting*** |
| *#*11 RCT OR randomized controlled trial OR clinical trial OR controlled trial (Setting: include all settings) |
| #12 effect OR efficacy OR therapy |
| *#13 #*11 *AND #*12 |
| *#14 #1 AND #5 AND #8 AND #9 AND #10 AND #13* |
| *#15* Remove *duplicates* |
| *#16* Limits: *2000-2019; English; adult; full report* |

**Appendix S2**. Between-group effect sizes of robot-assisted therapy on outcomes

| Outcome | Study | Instrument | Between-group effect size in terms of mean difference [95%CI] | | | |
| --- | --- | --- | --- | --- | --- | --- |
|  |  |  | Post-treatment | < 3 months post-treatment | 3-7 months post-treatment | >7 months  post-treatment |
| Motor control | Barker 2017 | MAS6^†^ | ^--^ | ^--^ | ^--^ | ^--^ |
|  | Daunoravience 2018 | FMA | 0.20 [-0.48, 0.87] | ^--^ | ^--^ | ^--^ |
|  | Dehem 2019 | FMA-Upper extremity | 0.29 [-0.29, 0.88] | ^--^ | 0.44 [-0.14, 1.04] | ^--^ |
|  | Hesse 2014 | FMA | -0.30 [-0.86, 0.26] | -0.25 [-0.80, 0.31] | ^--^ | ^--^ |
|  | Masiero 2014 | FMA-Total score^†^ | ^--^ | ^--^ | ^--^ | ^--^ |
|  |  | FMA-SEC^†^ | ^--^ | ^--^ | ^--^ | ^--^ |
|  |  | FMA-Wrist and hand^†^ | ^--^ | ^--^ | ^--^ | ^--^ |
|  | Orihuela Espina 2016 | FMA-Hand | 0.58 [-0.40,1.56] | ^--^ | ^--^ | ^--^ |
|  | Sale 2014 | FMA | **0.75 [0.19, 1.31]**** | ^--^ | ^--^ | ^--^ |
|  | Volpe 2000 | FMA-SEC | 0.56 [-0.03, 1.10] | ^--^ | ^--^ | ^--^ |
|  |  | FMA-Wrist and hand | 0.35 [-0.18, 0.88] | ^--^ | ^--^ | ^--^ |
|  | Wolf 2015 | FMA | 0.01 [-0.40, 0.42] | ^--^ | ^--^ | ^--^ |
| Functional independence | Daunoravience 2018 | FIM-Self care | 0.66 [-0.04, 1.35] | ^--^ | ^--^ | ^--^ |
|  | Dehem 2019 | Abilhand | -0.02 [-0.60, 0.57] | ^--^ | 0.31 [-0.27, 0.90] | ^--^ |
|  |  | Activlim | -0.02 [-0.60, 0.57] | ^--^ | 0.18 [-0.40, 0.77] | ^--^ |
|  | Hesse 2014 | BI | 0.27 [-0.29, 0.83] | 0.23 [-0.33, 0.79] | ^--^ | ^--^ |
|  | Masiero 2014 | FAT^†^ | ^--^ | ^--^ | ^--^ | ^--^ |
|  |  | FIM-motor^†^ | ^--^ | ^--^ | ^--^ | ^--^ |
|  | Stinear 2014 | MRS^‡^ | ^--^ | ^--^ | ^--^ | ^--^ |
|  | Villafane 2018 | BI | 0.10 [-0.59, 0.79] | ^--^ | ^--^ | ^--^ |
|  | Volpe 2000 | FIM-Motor | **1.19 [0.62, 1.77]**** | ^--^ | ^--^ | ^--^ |
|  |  | FIM-Cognition | **1.82 [1.11, 2.53]**** | ^--^ | ^--^ | ^--^ |
| Upper extremity performance | Dehem 2019 | WMFT | 0.24 [-0.35,0.83] | ^--^ | 0.40 [-0.18, 0.99] | ^--^ |
|  | Hesse 2014 | ARAT | -0.39 [-0.96, 0.17] | -0.47 [-1.05, 0.08] | ^--^ | ^--^ |
|  | Stinear 2014 | ARAT^¶^ |  | ^--^ | ^--^ | ^--^ |
|  | Villafane 2018 | QuickDASH | 0.33 [-0.36, 1.03] | ^--^ | ^--^ | ^--^ |
|  | Wolf 2015 | ARAT | -0.01 [0.42, 0.40] | ^--^ | ^--^ | ^--^ |
|  |  | WMFT | -0.02 [-0.43, 0.39] | ^--^ | ^--^ | ^--^ |
| Muscle tone | Barker 2017 | MAS^†^ | ^--^ | ^--^ | ^--^ | ^--^ |
|  | Daunoravience 2018 | MAS^§^ | ^--^ | ^--^ | ^--^ | ^--^ |
|  | Hesse 2014 | MAS | -0.06 [-0.62,0.49] | -0.10 [-0.65, 0.46] | ^--^ | ^--^ |
|  | Masiero 2014 | MAS^†^ | ^--^ | ^--^ | ^--^ | ^--^ |
|  | Sale 2014 | MAS-Elbow | 0.21 [-0.75, 0.33] | ^--^ | ^--^ | ^--^ |
|  |  | MAS-Shoulder | -0.41 [-0.95, 0.14] | ^--^ | ^--^ | ^--^ |
|  | Villafane 2018 | MAS | 0.26 [-0.43, 0.96] | ^--^ | ^--^ | ^--^ |
| Quality of life | Barker 2017 | SIS | 0.34 [-0.26, 0.95] | ^--^ | -0.29 [-0.99, 0.41] | -0.41 [-1.15, 0.33] |
|  | Dehem 2019 | SIS | -0.03 [-0.62, 0.55] | ^--^ | 0.42 [-0.17,1.02] | ^--^ |
|  | Stinear 2014 | SIS | -0.15 [-0.67, 0.37] | ^--^ |  | ^--^ |

Abbreviations: ACE-R, Addenbrooke Cognitive Examination-Revised; Active ROM, Active Range of Motion; ARAT, Action Research Arm Test; BBT, Box and Block Test; BI, Barthel Index; FAT, Frenchay Arm Test; FIM, Functional Independence Measurement; FMA, Fugl-Meyer Assessment; HAM-A, Hamilton Rating Scale for Anxiety; HAM-D, Hamilton Rating Scale for Depression; MAL, Motor Activity Log-28; MAS, Modified Ashworth Scale; MAS, Motor Assessment Scale; MI, Motricity Index; MP, Motor Power Scale; MRC, Medical Research Council, MRS, Modified Rankin Scale; MS, Motor Status Score; NIHSS, the National Institutes of Health Stroke Scale; pROM, passive Range of motion; QuickDASH, short version of the Disabilities of the Arm, Shoulder and Hand; RAI, Ritchie Articular Index; RCT, randomized controlled trial; RT, Robot-assisted therapy; SIS, Stroke Impact Scale; VAS, Visual Analog Scale; WFMT, Wolf Motor Function Test

**p* < .05, ***p* < .01, ****p* < .001

^†^Only median and inter-quartile range were reported.

^‡^Only median and range was reported.

^§^Only frequencies were reported.

^¶^Only odds ratio and 95%CI were reported.

**Appendix S3.** Summary of findings (SoF)

| **Robotic-assisted therapy compared to usual care for patients with sub-acute stroke** | | | | | | |
| --- | --- | --- | --- | --- | --- | --- |
| **Patient or population:** Patients with sub-acute stroke **Setting:** Stroke unit **Intervention:** Robot-assisted therapy (RT) **Comparison:** Usual care | | | | | | |
| **Outcomes** | **Anticipated absolute effect^*^ (95% CI)** | | **Effect size (95% CI)** | **No of participants (studies)** | **Certainty of the evidence (GRADE) **** | **Comments** |
|  | **Assumed Risk** | **Corresponding risk** |  |  |  |  |
|  | **Usual care** | **Robot-assisted therapy** |  |  |  |  |
| **Motor control**  assessed with:  Fugl-Meyer Assessment (FMA) Scale from: 0 to 66  Higher score indicates better motor control | The mean FMA score in the control groups was 36.4^1^ | The mean FMA in the intervention groups was 3.15 higher (2.80 lower to 8.93 higher) | 0.18 [-0.16,0.51] | 274 (5 RCTs) | ⊕⊕⊝⊝ LOW ^2 4^ | RT *may* results in little to no difference in motor function compared to usual care |
| **Functional independence**  assessed with:  Functional Independence Measure (FIM), Scale from: 13 to 91  Higher score indicates better functional independence | The mean FIM score in the control group was 43 points ^3^ | The mean FIM in the intervention groups was 3.8 higher (1.52 lower to 9.02 higher) | 0.4 [-0.16, 0.95] | 183 (4 RCTs) | ⊕⊕⊝⊝ LOW ^2 5^ | RT *may* result in little to no difference in functional independence compared to usual care |
| **Upper extremity performance**  assessed with: Action Research Arm Test  Scale from: 0-57 | The mean ARAT score in the control group was 30.1 points ^6^ | The mean ARAT in the intervention group was 0.39 higher (10.8 lower to 11.6 higher) | 0.01 [-0.28,0.30] | 219 (4 RCTs) | ⊕⊕⊕⊝ MODERATE ^2^ | RT *properly* result in little to no difference in upper extremity performance compared to usual care |
| **Muscle tone**  assessed with:  Modified Ashworth Scale (MAS)  Scale from: 0-4 | The mean MAS score in the control group was 1.24 points ^7^ | The mean MAS in the intervention group was 0.04 lower (0.26 lower to 0.29 higher) | -0.04 [-0.38,0.3] | 135 (3 RCTs) | ⊕⊕⊝⊝ LOW^2 5^ | RT *may* result in little to no difference in muscle tone compared to usual care |
| **Quality of life**  assessed with:  Stroke Impact Scale (SIS)  Scale from: 0-100 | The mean SIS score in the control group was 53.1 points ^8^ | The mean SIS in the intervention group was 0.66 higher (6.6 lower to 7.92 higher) | 0.03[-0.3,0.36] | 149 (3 RCTs) | ⊕⊕⊕⊝ MODERATE^5^ | RT *probably* results in little to no difference in quality of life compared to usual care |
| **Adverse events** | All studies reported no major side effects/ complications. | | - |  | ⊕⊕⊕⊕ HIGH |  |
| **Costs** | One study reported that the cost of RT and individual arm therapy per session for each patient was 4.15 € and 10.00 €, respectively. | | - | 50 (1 RCT) | ⊕⊕⊝⊝ LOW ^9^ |  |
| *The risk in the intervention group (and its 95% confidence interval) is based on the assumed risk in the comparison group and the relative effect of the intervention (and its 95% CI). To calculate the anticipated absolute effect of an estimate, the standardized mean difference (SMD) generated from the pooled data was re-expressed into mean difference (MD) for a better presentation as units of a familiar measure. This approach was carried out by calculating the absolute difference (together with 95% confidence interval, CI) in means by multiplying the SMD with an estimate of the SD associated with the instrument that is the most commonly used across the included studies, in which this SD was calculated by the weighted average of post-treatment SDs across all intervention groups of the included studies that used the selected instrument (Higgins & Green, 2011).  ** The overall quality of evidence was rated as high, moderate, low, or very low. If the included randomized controlled trials had limitations in study limitations, consistency of effect, imprecision, indirectness, and publication bias, the quality rating of evidence was downgraded by one (for serious limitations) or two levels (for very serious limitations). **CI:** Confidence interval; **RR:** Risk ratio; **OR:** Odds ratio; | | | | | | |
| **GRADE Working Group grades of evidence** **High certainty:** We are very confident that the true effect lies close to that of the estimate of the effect **Moderate certainty:** We are moderately confident in the effect estimate: The true effect is likely to be close to the estimate of the effect, but there is a possibility that it is substantially different **Low certainty:** Our confidence in the effect estimate is limited: The true effect may be substantially different from the estimate of the effect **Very low certainty:** We have very little confidence in the effect estimate: The true effect is likely to be substantially different from the estimate of effect | | | | | | |

^1^ This range of score was based on five included studies that measured motor control by using the Fugl-Meyer Assessment (FMA) in their control groups (Daunoraviciene, Adomaviciene, Grigonyte, Griskevicius, & Juocevicius, 2018; Dehem et al., 2019; Hesse, Hess, Werner, Kabbert, & Buschfort, 2014; Sale et al., 2014; Wolf et al., 2015).

^2^ Some of the studies were at high/ unclear risk of bias due to the lack of blinding of outcome assessors and/or insufficient information on allocation concealment. The quality was downgraded by 1 point.

^3^ The mean scores were obtained from Volpe et al (2000) that measured functional independence by using the Functional Independence Measure (FIM) (Volpe et al., 2000).

^4^ The results were based on wide 95% confidence intervals. The quality was downgraded by 1 point.

^5.^ The results were based on studies with small sample sizes (16-31 per arm). The quality of evidence was downgraded by 1 point.

^6^ The mean scores were obtained from Hesse et al (2014) and Wolf et al (2015) that measured upper extremity performance by using the Action Research Arm Test (ARAT) (Hesse et al., 2014; Wolf et al., 2015)

^7^ The mean scores were obtained from three included studies that measured muscle tone by using the Modified Ashworth Scale (MAS) (Hesse et al., 2014; Sale et al., 2014; Villafane et al., 2018).

^8^ The mean scores were obtained from three included studies that measured quality of life by using the Stroke Impact Scale (Barker, Hayward, Carson, Lloyd, & Brauer, 2017; Dehem et al., 2019; Stinear, Petoe, Anwar, Barber, & Byblow, 2014)

^9^The results were only based on one study. The quality was downgraded by 2 points.

**References**

Barker, R. N., Hayward, K. S., Carson, R. G., Lloyd, D., & Brauer, S. G. (2017). SMART Arm training with outcome-triggered electrical stimulation in subacute stroke survivors with severe arm disability: a randomized controlled trial. *Neurorehabilitation and Neural Repair, 31*(12), 1005-1016. doi:10.1177/1545968317744276

Daunoraviciene, K., Adomaviciene, A., Grigonyte, A., Griskevicius, J., & Juocevicius, A. (2018). Effects of robot-assisted training on upper limb functional recovery during the rehabilitation of poststroke patients. *Technology and Health Care, 26*(S2), 533-542. doi:10.3233/thc-182500

Dehem, S., Gilliaux, M., Stoquart, G., Detrembleur, C., Jacquemin, G., Palumbo, S., . . . Lejeune, T. (2019). Effectiveness of upper-limb robotic-assisted therapy in the early rehabilitation phase after stroke: a single-blind, randomised, controlled trial. *Annals of Physical and Rehabilitation Medicine, 62*(5), 313-320. doi:10.1016/j.rehab.2019.04.002

Hesse, S., Hess, A., Werner, C. C., Kabbert, N., & Buschfort, R. (2014). Effect on arm function and cost of robot-assisted group therapy in subacute patients with stroke and a moderately to severely affected arm: a randomized controlled trial. *Clinical Rehabilitation, 28*(7), 637-647. doi:10.1177/0269215513516967

Higgins, J. P., & Green, S. (2011). *Cochrane Handbook for Systematic Reviews of Interventions* (Vol. 4). Chichester, United Kingdom: John Wiley & Sons Ltd.

Sale, P., Franceschini, M., Mazzoleni, S., Palma, E., Agosti, M., & Posteraro, F. (2014). Effects of upper limb robot-assisted therapy on motor recovery in subacute stroke patients. *Journal of Neuroengineering and Rehabilitation, 11*, 104. doi:10.1186/1743-0003-11-104

Stinear, C. M., Petoe, M. A., Anwar, S., Barber, P. A., & Byblow, W. D. (2014). Bilateral priming accelerates recovery of upper limb function after stroke: a randomized controlled trial. *Stroke, 45*(1), 205-210. doi:10.1161/strokeaha.113.003537

Villafane, J. H., Taveggia, G., Galeri, S., Bissolotti, L., Mulle, C., Imperio, G., . . . Negrini, S. (2018). Efficacy of short-term robot-assisted rehabilitation in patients with hand paralysis after stroke: a randomized clinical trial. *Hand, 13*(1), 95-102. doi:10.1177/1558944717692096

Volpe, B. T., Krebs, H. I., Hogan, N., Edelstein, O. L., Diels, C., & Aisen, M. (2000). A novel approach to stroke rehabilitation: robot-aided sensorimotor stimulation. *Neurology, 54*(10), 1938-1944. doi:10.1212/wnl.54.10.1938

Wolf, S. L., Sahu, K., Bay, R. C., Buchanan, S., Reiss, A., Linder, S., . . . Alberts, J. (2015). The HAAPI (Home Arm Assistance Progression Initiative) Trial: a novel robotics delivery approach in stroke rehabilitation. *Neurorehabilitation and Neural Repair, 29*(10), 958-968. doi:10.1177/1545968315575612
